# Serosurvey of Anti-*Toxoplasma gondii* Antibodies in Homeless Persons of São Paulo City, Southeastern Brazil

**DOI:** 10.3389/fpubh.2020.580637

**Published:** 2020-11-05

**Authors:** Laís Giuliani Felipetto, Pedro Irineu Teider-Junior, Felipe Fortino Verdan da Silva, Ana Carolina Yamakawa, Louise Bach Kmetiuk, Anahi Chechia do Couto, Camila Marinelli Martins, Eduarda Stankiwich Vaz, Leila Sabrina Ullmann, Helio Langoni, Jorge Timenetsky, Andrea Pires dos Santos, Alexander Welker Biondo

**Affiliations:** ^1^Department of Veterinary Medicine, Graduate College of Veterinary Science, Federal University of Paraná, Curitiba, Brazil; ^2^Clinical Analysis Laboratory Unit, Clinics Hospital, Federal University of Paraná, Curitiba, Brazil; ^3^Department of Veterinary Hygiene and Public Health, São Paulo State University, Botucatu, Brazil; ^4^Graduate College of Cellular and Molecular Biology, Federal University of Paraná, Curitiba, Brazil; ^5^Department of Nursing and Public Health, State University of Ponta Grossa, Ponta Grossa, Brazil; ^6^AAC&T Research Consulting LTDA, Curitiba, Brazil; ^7^Institute of Biotechnology, São Paulo State University (UNESP), Botucatu, Brazil; ^8^Department of Medical Microbiology, University of São Paulo, São Paulo, Brazil; ^9^Department of Comparative Pathobiology, Purdue University, West Lafayette, IN, United States; ^10^Department of Veterinary Medicine, Federal University of Paraná, Curitiba, Brazil

**Keywords:** homeless, *Toxoplasma gondii*, HIV, vulnerability, serology

## Abstract

Seroprevalence of *Toxoplasma gondii* has been extensively studied in a variety of different human populations. However, no study has focused on homeless populations. Accordingly, the present study aimed to assess the seroprevalence of anti-*T. gondii* antibodies and the risk factors associated in homeless persons from homeless shelter of São Paulo city, southeastern Brazil. In addition, anti-HIV antibodies and associated risk of *T. gondii* and HIV coinfection have been evaluated. Anti-*T. gondii* antibodies were detected by indirect fluorescent antibody test. In addition, anti-HIV levels were tested by chemiluminescence enzyme immunoassay, with positive samples confirmed by rapid immunoblot assay. Overall, IgG anti-*T. gondii* seropositivity was found in 43/120 (35.8%) homeless persons, with endpoint titers varying from 16 to 1,024. The only two pregnant women tested were negative for IgM by chemiluminescence enzyme immunoassay, with normal parturition and clinically healthy newborns in both cases. There were no statistical differences in the risk factors for anti-*T. gondii* serology (*p* > 0.05). Anti-HIV seropositivity was found in 2/120 (1.7%) homeless persons, confirmed as HIV-1. One HIV seropositive individual was also sero-reactive to IgG anti-*T. gondii*, and both were negative to IgM anti-*T. gondii*. This is the first study that reports the serosurvey of *T. gondii* in homeless persons worldwide. Despite the limited sample size available in the present study, our findings have shown that the prevalence of anti-*T. gondii* antibodies in homeless persons herein was lower than the general population, probably due to homeless diet habit of eating mainly processed food intake. No statistical differences were found regarding risk factors for anti-*T. gondii* exposure in homeless persons. Future studies should be conducted to fully establish risk factors for anti-*T. gondii* exposure in homeless persons.

## Introduction

Homeless persons have been described as one of the three most vulnerable populations, along with refugees and incarcerated persons (1). Morbidity and mortality of diseases have been reportedly higher in homeless than general population, probably due to social inequality associated with lack of settled home, job opportunity, and a series of family problems including drug addiction, mental health disorders, and social justice issues, mostly exacerbated by absence of health assistance (2). A population of 1.6 billion people without adequate housing has been estimated worldwide, of which 100 million are homeless (3, 4). In Brazil, the nationwide homeless population has been estimated in 101,854 individuals, with about 40.1% living in cities with more than 900,000 inhabitants and about 16,000 living on streets of São Paulo city (5, 6).

*Toxoplasma gondii* is a coccidian parasite relying on cats and other family Felidae as definitive hosts, which may shed fecal oocysts infecting a variety of homeothermic intermediate hosts (7). Human infection has been typically subclinical or asymptomatic, with the time of infection and transmission route not known in most cases (8, 9). Despite that, in immunodeficient people, such as in HIV-toxoplasmosis combination, the protozoan can cause severe clinical manifestations, with invasion into the central nervous system and encephalitis (10, 11).

The human *T. gondii* seroprevalence has been extensively reported, ranging from 0.8 to 77.5% worldwide (12), few reports are available for vulnerable populations including 123/597 (20.6%) aboriginal individuals of Thailand, 236/628 (37.6%) prisoners of Turkey, mostly (92.8%) males, and 63/199 (31.7%) pregnant refugee and borderline migrant women of Asia (13–15). In Brazil, seropositivity of *T. gondii* has been ranged from 14/65 (21.5%) urban students of northeastern region to 113/116 (97.4%) farmers of dairy cattle farm in central-western region (16, 17). In vulnerable populations of Brazil, the positivity reported was 131/231 (56.7%) inhabitants living in riverside communities of northern region and 119/148 (80.4%) indigenous of central-western region (18, 19).

Although *T. gondii* seroprevalence has been reported in others vulnerable populations, no study has focused on homeless populations. Accordingly, the present study aimed to assess the seroprevalence of anti-*T. gondii* and the associated risk factors for exposure in homeless persons from homeless shelter of São Paulo city, southeastern Brazil. In addition, anti-HIV antibodies and associated risk of *T. gondii* and HIV coinfection have been evaluated.

## Materials and Methods

### Local of Study

The present study represents a descriptive cross-sectional seroepidemiological approach of the homeless population from the western São Paulo city (23°33′1″S, 46°38′2″W) shelter (Social Center “Our Lady of Good Delivery”), responsible for daytime attendance of all the city region. The shelter is a Non-Governmental Organization, sponsored by a city partnership daily attending around 800–1,200 homeless persons, providing meals, medical assistance, job opportunities, and recreational activities.

São Paulo city, capital of São Paulo State, southeastern Brazil, has been ranked as the most populated city of Latin America with 11,253,500 people and the tenth-largest Gross Domestic Product (GDP) of the world, with a very high Human Development Index (HDI) (0.805). The city is located under a humid subtropical climate with average temperatures ranging from 19°C (winter) to 25°C (summer) (20).

The present study was conducted along with the city multi-task professionals team at the São Paulo City Secretary of Health, called “street outreach office,” which includes physicians, nurses, dentists, social assistants, and psychologists, based on the strategy of the Brazilian Unified Health System (21). This city official team offers permanent assistance and save clinical records of the homeless population, promoting health actions on a continuing care bond.

### Epidemiological Data Collection

Epidemiological analyses were performed based on a questionnaire associated with homeless persons exposure to *T. gondii* and HIV, which included: (1) Demographic profile: sex, marital status, racial self-declaration, age, educational background, income, and city of origin; (2) Social profile: travel to other cities, communication with family, causes for becoming homeless, homelessness time, resting place, have children, have own children, pregnant woman, live alone, pet owner, use of licit and illicit drugs, alcohol consumption, tobacco use, marijuana use, cocaine use, crack use, assistance by the Psychosocial Care Centers (CAPS) as part of the free national Unified Health System; (3) Hygiene profile: bath frequency, change of clothes frequency, wash clothes, body lice (*Pediculus humanus humanus*) bites, and body lice presence (Supplementary Material). Refusal to fully or partially answer any question or incomplete answers were accepted and registered.

### Sample Collection

Blood samples of homeless persons were conveniently drawn from June to August 2018, which was the limited timeframe permission issued by the City Secretary of Health at the time. A minimal sampling of 71 individuals was calculated using commercially available software (Epi Info 7.7.7.6) based on an estimative of 16,000 homeless persons in São Paulo City and homeless HIV infection prevalence of 4.9% (22), the sampling was simple with 95% confidence and 5% accuracy. Homeless persons were recruited by government health officials and invited to participate voluntarily of research, and blood collection was performed by cephalic puncture. Samples were placed in tubes without anticoagulant and kept at 25°C until visible clot retraction. Serum was separated by centrifugation at 3,000 revolutions per minute for 10 min and stored at −20°C until processing.

In addition, the packed cell volume (PCV) by capillary tube centrifugation and total plasma protein (TPP) by refractometry were performed on the day of sampling and immediately given to the shelter administration. Due to the shelter demand, homeless persons were also examined for body lice (*Pediculus humanus humanus*) bites and presence, as previously described at the same shelter (23). The University made a clothing donation drive during the study and researchers offered clean clothes to all lice-infested homeless persons.

### Serological Diagnosis

Detection of *T. gondii* antibodies was performed by indirect immunofluorescent antibody test (IFAT) (24), with serial serum dilutions of 1:16–1:4,096 performed in pH 7.2 phosphate-buffered saline solution (PBS) with the cut-off titer of ≥16 IU. Immunofluorescence slides were previously sensitized with 0.1% formaldehyde to inactivated tachyzoites of *T. gondii* (RH strain) obtained from an intraperitoneal lavage in Swiss mice after 3 days of inoculation. A commercial anti-human IgG antibody, conjugated with fluorescein isothiocyanate (Bethyl Laboratories, Montgomery, TX, USA) was used as secondary antibody. For positive samples, the highest titer was considered with at least 50% of fluorescence on the border of tachyzoites.

In addition, the detection of HIV was performed by chemiluminescent microparticle immunoassay (CMIA) (Alinity's HIV Ag/Ab Combo Reagent Kit, Abbott Laboratories, Chicago, IL, USA) used for the simultaneous qualitative detection of HIV p24 antigen and antibodies to HIV type 1 (HIV-1 group M and group O) and/or type 2 (HIV-2) in human serum. The resulting chemiluminescent reaction was measured as relative light units (RLU). Cases of reactive serology were confirmed by a commercially available rapid immunoblot assay (DPP HIV1/2®, Fiocruz, Rio de Janeiro, Brazil). Samples of the pregnant woman and the HIV-positive individuals were tested for *T. gondii* IgM presence by CMIA (Anility's Toxo IgM Reagent Kit, Abbott Laboratories, Chicago, IL, USA).

### Statistical Analysis

Statistical analysis was performed using SPSS 20.0 (25). Frequencies of *T. gondii* and HIV seropositivity (absolute and relative) were determined by the stratification of the observations according to demographic, social, and hygiene profiles. The Chi-Square test was used to determine the bivariate association between studied variables, and odds ratios (OR) were used for the association of *T. gondii* prevalence and potential risk factors.

## Results

Overall, anti-*T. gondii* antibodies were detected in 43/120 (35.8%, CI 95% 26.7–43.0%) homeless persons, with endpoint titers varying from 16 to 1,024. No statistical differences were found regarding risk factors for anti-*T. gondii* exposure (*p* > 0.05) in homeless persons (Table 1).

**Table 1:** Statistical results of univariate and multiple logistic regression models of associated risk factors for seropositivity of IgG anti-*T. gondii* antibodies in homeless persons.

**Risk factor**		**Total**		**Positive**		**Negative**		***p*-value**	**OR**
		***N***	**% of total**	***N***	**% of line**	***N***	**% of line**		
***T. gondii***									
**1) DEMOGRAPHIC PROFILE**									
Sex	Male	107	89.2	41	38.3	66	61.7	0.282	0.48 (0.12–1.85)
	Female	13	10.8	3	23.1	10	76.9		
Marital status	Unmarried	108	90.0	41	38.0	67	62.0	0.377	0.54 (0.13–2.12)
	Accompanied	12	10.0	3	25.0	9	75.0		
Racial self-declaration	White	28	23.3	8	28.6	20	71.4	0.310	1.60 (0.64–4.03)
	Non-white	92	76.7	36	39.1	56	60.9		
Educational background	None to 8th grade	91	75.8	34	37.4	57	62.6	0.438	0.72 (0.32–1.64)
	High School and University	29	24.2	9	31.0	20	69.0		
Income	No income	100	84.7	37	37.0	63	63.0	0.879	1.08 (0.38–3.03)
	With income	18	15.3	7	38.9	11	61.1		
City of origin	São Paulo city	38	31.9	12	31.6	26	68.4	0.404	1.41 (0.62–3.02)
	Other cities	81	68.1	32	39.5	49	60.5		
Travel to other cities	Yes	23	20.2	9	39.1	14	60.9	0.799	1.13 (0.44–2.89)
	No	91	79.8	33	36.3	58	63.7		
**2) SOCIAL PROFILE**									
Contact with family	Yes	66	55.5	23	34.8	43	65.2	0.582	1.22 (0.58–2.59)
	No	53	44.5	21	39.6	32	60.4		
Causes for becoming homeless	Alcohol and drugs	28	23.3	9	32.1	19	67.9	0.736	1.16 (0.47–2.89)
	Family conflicts	48	40.0	19	39.6	29	60.4	0.360	0.69 (0.32–1.51)
	Housing loss	13	10.8	3	23.1	10	76.9	0.347	1.89 (0.49–7.33)
	Other	18	15.0	9	50.0	9	50.0	0.140	0.47 (0.17–1.30)
	Unemployment	34	28.3	10	29.4	24	70.6	0.433	1.41 (0.59–3.35)
Resting place	Hostel	68	45.3	26	38.2	42	61.8	0.683	0.85 (0.40–1.81)
	Street	52	34.7	18	34.6	34	65.4	0.905	1.05 (0.43–2.54)
	Occupancy	30	20	10	33.3	20	66.7	0.662	1.21 (0.50–2.90)
Pregnant woman	Yes	2	1.7	0	0.0	2	100.0	0.278	^*^
	No	118	98.3	44	37.3	74	62.7		
Have children	Yes	81	67.5	33	40.7	48	59.3	0.182	0.57 (0.25–1.30)
	No	39	32.5	11	28.2	28	71.8		
Have own children	Live together	4	5.1	0	0.0	4	100.0	0.087	^*^
	Other people	74	94.9	32	43.2	42	56.8		
Live alone	Yes	52	46.0	19	36.5	33	63.5	0.815	0.91(0.42–1.97)
	No	61	54.0	21	34.4	40	65.6		
Pet owner	Yes	26	22.4	12	46.2	14	53.8	0.231	0.58 (0.24–1.41)
	No	90	77.6	30	33.3	60	66.7		
Dog owner	Yes	21	18.1	11	52.4	10	47.6	0.088	0.44 (0.16–1.14)
	No	95	81.9	31	32.6	64	67.4		
Cat owner	Yes	6	5.2	2	33.3	4	66.7	0.880	1.14 (0.20–6.52)
	No	110	94.8	40	36.4	70	63.6		
Use of licit and/or illicit drugs	Yes	91	75.8	35	38.5	56	61.5	0.470	0.72 (0.29–1.75)
	No	29	24.2	9	31.0	20	69.0		
Alcohol consumption	Yes	52	43.3	18	34.6	34	65.4	0.471	0.76 (0.36–1.60)
Tobacco use	Yes	32	26.7	16	50.0	16	50.0	0.068	0.46 (0.20–1.06)
Marijuana use	Yes	31	25.8	12	38.7	19	61.3	0.784	0.88 (0.38–2.06)
Cocaine use	Yes	34	28.3	12	35.3	22	64.7	0.844	1.08 (0.47–2.48)
Crack use	Yes	16	13.3	7	43.8	9	56.2	0.528	0.71 (0.24–2.06)
Other drugs	Yes	5	4.2	3	60.0	2	40.0	0.269	0.36 (0 05–2.30)
Assistance by Psychosocial Care Centers (CAPS)	Yes	31	25.8	10	32.3	21	67.7	0.554	1.29 (0.54–3.08)
	No	89	74.2	34	38.2	55	61.8		
**3) HYGIENE PROFILE**									
Bath frequency	Daily	99	82.5	34	34.3	65	65.7	0.252	1.73 (0.67–4.50)
	Sporadic	21	17.5	10	47.6	11	52.4		
Wash clothes	Yes	82	68.3	30	36.6	52	63.4	0.978	1.01 (0.45–2.24)
	No	38	31.7	14	36.8	24	63.2		
Change clothes frequency	Daily	50	42.7	17	34.0	33	66.0	0.838	1.08 (0.50–2.33)
	Sporadic	67	57.3	24	35.8	43	64.2		
Body lice bite	Yes	63	59.4	23	36.5	40	63.5	0.752	1.13 (0.51–2.52)
	No	43	40.6	17	39.5	26	60.5		
Presence of body lice	Yes	17	14.2	4	23.5	13	76.5	0.225	2.06 (0.62–6.77)
	No	103	85.8	40	38.8	63	61.2		

* *The percentages can go higher than 100% because individuals could answer more than one option*.

Associated risk factors for the presence of anti-*T. gondii* were not statistically significant regarding educational background (*p* = 0.438), income (*p* = 0.805), resting place (hostels, street, occupancy) (*p* > 0.05), pregnancy (*p* = 0.567), pet owner (*p* = 0.399); cat owner (*p* = 0.916), bath frequency (*p* = 0.652), age (*p* = 0.223), and homelessness time (*p* = 0.827) (Table 2). The homeless persons sampled were mostly men counting 107/120 (89.2%) individuals, with 39/107 (36.8%) seropositive samples for *T. gondii*. On the other side, women accounted for 13/120 (10.8%) with 3/13 (23.1%) positive samples. Despite in lower number, eight women were within the reproductive age of 24–35 years old, and 7/8 (87.5%) presented negative serology for *T. gondii*, including the two pregnant homeless women exposed to infection. Fortunately, the two pregnant women tested negative for anti-*T. gondii* antibodies in both IgG by IFAT and IgM by CMIA, with normal parturition and clinically healthy newborns in both cases.

**Table 2:** Average, median, and standard deviation (SD) of *T. gondii* positive and negative homeless persons according to age (years), homelessness time (months), number of children, number of dogs, number of cats, packed cell volume (PCV), total plasma protein (TTP).

**Risk factor**	**Negative**			**Positive**			***p*-value**
	**Average**	**Median**	**SD**	**Average**	**Median**	***SD***	
***T. gondii***							
Age (years)	43.55	44.50	14.03	41.18	42.50	11.96	0.412
Homelessness time (months)	67.68	36.00	78.43	85.10	36.00	103.60	0.557
Number of children	2.19	1.00	4.02	2.07	2.00	2.08	0.385
Number of dogs	0.34	0.00	1.27	0.62	0.00	1.40	0.081
Number of cats	0.05	0.00	0.23	0.17	0.00	0.82	0.928
PCV	42.46	42.00	4.00	42.84	44.00	4.40	0.629
TPP	7.65	7.60	0.63	7.64	7.60	0.52	0.918

In addition, a total of 2/120 (1.7%, CI 95% 0.0–4.2%) anti-HIV seropositive homeless persons were detected by CMIA and confirmed by rapid immunoblot assay tests. No evaluation of HIV risk factors was made due to low seropositive rate.

## Discussion

To the authors' knowledge, this is the first study that reports the serosurvey of *T. gondii* in homeless persons and the associated risk factors.

The seroprevalence of anti- *T. gondii* antibodies herein (35.8%) was higher than other vulnerable populations, such as aborigines (20.6%), pregnant refugee, and borderline migrant women (31.7%), but similar to incarcerated populations (37.6%) (13–15). In the present study, a total of 91/114 (79.8%) homeless sampled herein declared not recently traveling to other cities, although 81/119 (68.1%) have been born or previously lived another city or region.

In Brazil, the anti-*T. gondii* seroprevalence herein was higher than the general population of the northeastern region, with 14/65 (21.5%) seropositive urban students, but lower than the central-western region, with 113/116 (97.4%) farmers from a single dairy cattle farm with domestic cats and potentially contaminated environment (16, 17). Interestingly, the seroprevalence of anti-*T. gondii* antibodies in the present study was lower than other Brazilian neglected populations, such as 131/231 (56.7%) persons of riverside communities in the northern and 119/148 (80.4%) indigenous persons in the central-western region (18, 19). In São Paulo, similar results were found, with 110/339 (32.4%) seropositive children from a low-socioeconomic community (26). Not surprisingly, a previous study has shown an association between high seropositivity for *T. gondii* and socioeconomic vulnerability in southern Brazil, with 526/715 (73.6%) seropositive individuals, particularly in low-income families (27).

Although low education and socioeconomic status have been associated with increased risk of *T. gondii* infection in different Brazilian studies (28–30), no statistical association with *T. gondii* infection was previously found regarding educational background and income, probably due to the broadly variable classification of and the low population homogeneity (31, 32). Similarly, no association was found in either education or income, likely associated to the impact of the vulnerable living style, with mostly drug addicts with poor eating habits.

Since the low socioeconomic status may be associated to malnutrition and might impair the host defense against protozoan infection, the relatively low seroprevalence of anti- *T. gondii* antibodies in homeless herein may be consequence of mainly consumption of ready-to-eat foods, as already indicated by previous studies on homelessness and food preparation facilities, which have reported dependence on charity meals, such as pre-prepared foods, processed foods or popular snacks (33–36). Not surprisingly, pre-processed ready-to-eat and meat-based foods have been shown to inactivate *T. gondii* cysts (37).

In addition, healthier and more expensive items, such as meat, fish, vegetables, and fruits have been less often consumed by homeless (33, 36, 38, 39), which may be a contributing factor to the low *T. gondii* seroprevalence found in this study. Hence, it is reasonable to speculate that the beneficial shelters, hostels, and meal services may have offered protection to the homeless population (36, 40) but not as nutritional good food habits when compared to the general population. Although no homeless person has been diagnosed with either anemia by packed cell volume (PCV) or hypoproteinemia by refractometry, such tests may not have enough sensitivity to detect chronic alimentary deficiencies, which should be further investigated.

A previous study with pregnant women has shown high seroprevalence of specific anti-*T. gondii* antibodies (68.4%; 333/487) and vertical transmission associated with social vulnerability in central Brazil (41). In the present study, despite negative for both IgG and IgM anti-*T. gondii* antibodies, the two pregnant women sampled, fortunately, gave birth to clinically healthy babies. *T. gondii* infection during pregnancy has been a significant problem, especially during the first months, and may result in spontaneous abortion, fetal and/or neonatal death or several congenital disabilities, such as hydrocephalus, central nervous system disorders, and chorioretinitis (42, 43). In the second and third trimester, newborns have usually been asymptomatic, with symptoms appearing late in childhood or early in adulthood, and may sporadically cause visual impairment (42, 44, 45). In addition, congenital toxoplasmosis may also be associated with reactivation of the chronical maternal infection, particularly in HIV-infected and immunosuppressed women (46). As 7/8 (87.5%) women herein were within reproductive age and presented negative serology for *T. gondii*, the homeless may be highly unprotected to infection during pregnancy.

Although the present study has shown no association between *T. gondii* infection and pet ownership, including stray cat owners, corroborating with previous studies in rural and other vulnerable populations of southern Brazil (27, 31), only 6/116 (5.2%) homeless persons owned a total of 11 cats. Outdoor lifestyle of stray cats may include hunting of birds and rodents, leading to raw meat dietary habits and increased risk of *T. gondii* ingestion (47). Nonetheless, human toxoplasmosis outbreaks may be attributed to exposure to infected cats, which may indicate an important role of cat oocyst excretion on infection spreading (48, 49), and homeless might be daily overexposed to environmental contamination. Another interestingly study has reported that 14/35 (40.0%) fresh vegetables and fruits collected from local producers and supermarket suppliers of Portugal and Spain were positive to *T. gondii* by PCR and microscopic autofluorescence (50). However, as mentioned above, homeless dietary habits of high intake of processed foods and low fresh meat, fish, vegetables and fruits may have led to lower *T. gondii* exposure. In fact, processing food methods, including high heating, curing, cooking, freezing, or chemical methods, have been shown to denature *T. gondii* proteins and remove or inactivated *T. gondii* oocysts, constituted by 90% cysteine and tyrosine (51).

Body lice (*Pediculus humanus humanus*) has been recently considered as a reemerging problem among homeless populations in France, Italy, USA, Colombia, and Brazil (23, 52–55). Even though body lice presence suggests social vulnerability and 17/120 (14.2%) of the homeless herein were infested with lice, no statistically risk of *T. gondii* exposure was found.

In addition to the *T. gondii* serosurvey, the HIV seroprevalence has been assessed herein. In the present study, 2/120 (1.7%) homeless persons was positive to anti-HIV antibodies, above the estimated prevalence of 0.4% for the general Brazilian population and lower than a recent study with 69/1,402 (4.9%) seropositive homeless (22, 56). Despite previous studies have shown a high prevalence of *T. gondii* and HIV co-infection, with 35.8% worldwide in a meta-analysis study and up to 88.4% of individuals coinfected in Ethiopia (57, 58), the analysis of associated risk factors herein has been impaired due to low HIV positive frequency.

One limitation of the present study was the relatively low number of samples, which was caused by the short timeframe sampling permission, associated to refusal of homeless persons in participating on the study. Difficulties in accessing such population may partially explain the lack of studies involving homeless and zoonoses, including toxoplasmosis. Although it may have generated insufficient data to provide strong basis for a statistical analysis, the results herein may still contribute on understanding homeless health and well-being. In addition, questionnaires to assess homeless information may be problematic, particularly regarding food intake and dietary habits, once such a population has often shown a chaotic lifestyle and a high prevalence of drug use and mental health disorders (36). Finally, further studies should be conducted in higher number and different homeless populations to fully establish the exactly impact of *T. gondii* in homeless persons worldwide.

In conclusion, this is the first study that reports the serosurvey of *T. gondii* in homeless persons worldwide. Despite the limited sample size available in the present study, our findings have shown that the frequency of anti-*T. gondii* antibodies in homeless persons was lower than the general population, probably due to homeless diet habit of eating mainly processed food intake. No statistical differences were found regarding risk factors for anti-*T. gondii* exposure in homeless persons. Future studies should be conducted to fully establish risk factors for anti-*T. gondii* exposure in homeless persons.

## Data Availability Statement

The original contributions presented in the study are included in the article/Supplementary Materials, further inquiries can be directed to the corresponding author/s.

## Ethics Statement

The studies involving human participants were reviewed and approved by the Ethics Committee in Human Research at the Federal University of Paraná (CAAE: 80099017.3.0000.0102, protocol number: 2.512.196), by the Ethics in Human Health Committee at the São Paulo City Secretary of Health (CAAE: 80099017.3.3004.0086, protocol number: 3.366.684) and by Ethics Committee in Human Research of the Clinics Hospital at the Federal University of Paraná (CAAE: 80099017.3.3005.0096, protocol number: 3.623.845), all subordinate to the National Human Ethics Research Committee of the Brazilian Ministry of Health. The Informed Consent Form was applied to all homeless persons, according to the ethical guidelines and principles of Federal University of Paraná. All participants research provided written informed consent. The patients/participants provided their written informed consent to participate in this study.

## Author Contributions

LF, PT-J, AY, LK, CM, LU, and AB: drafting and revision of the manuscript. LF, LU, HL, JT, AS, and AB: initiation, conception, design, and coordination of the research project. LF, FS, AC, CM, and EV: development of the intervention and evaluation materials. LF, PT-J, LK, CM, LU, AS, and AB: implementation of the intervention. All authors read and approved the final version of the manuscript.

## Conflict of Interest

CM is employed by the company AAC&T Research Consulting LTDA. The remaining authors declare that the research was conducted in the absence of any commercial or financial relationships that could be construed as a potential conflict of interest.
